# Height and height velocity of early/average/late maturing children and adolescents from longitudinal study of the Kangwha cohort

**DOI:** 10.1186/1687-9856-2015-S1-O27

**Published:** 2015-04-28

**Authors:** Hyun Wook Chae, Ah Reum Kwon, Jung Min Ahn, Dae Ryong Kang, Ha Yan Kim, Sun Min Oh, Hyeon Chang Kim, Il Suh, Duk Hee Kim, Ho-Seong Kim

**Affiliations:** 1 Department of Pediatrics, Yonsei University College of Medicine, Seoul, Korea; 2 Biostatistics Collaboration Unit, Yonsei University College of Medicine, Seoul, Korea; 3 Department of Preventive Medicine, Yonsei University College of Medicine, Seoul, Korea; 4 Sowha Children’s Hospital, Seoul, Korea

## Aims

The timing of the growth spurt and gender differences in physical growth can vary greatly, and it may contribute to the final height. However, there are few studies about height and height velocity of early/average/late maturing children because of the requirements of a population based longitudinal study. We investigated the height and height velocity according to growth tempo from the Kangwha cohort.

## Methods

The present study conducted as a part of a community-based prospective cohort study from 1986 to 1999 with 800 school children (359 males, 441 females). We calculated 2 standard deviation of peak height velocity (PHV) and the age of PHV, and then grouped the subjects into early/average/late maturing groups. We compared the results of 3 groups and investigated the differences.

## Results

The age at PHV was 12 in boys and 10 in girls, and height velocity at PHV was 8.62 cm/yr in boys and 7.07 cm/yr in girls on average tempo growth. In boys, the age of PHV was 11 and PHV 9.82 cm/yr in the early maturing group, and the age of 13 and 8.97 cm/yr in late maturing group. In girls, the age of PHV was 9 and PHV 9.75 cm/yr in the early maturing group, however, in the late maturing group; the difference was not significant compared with average tempo. The final height of each group was not different.

## Conclusion

Final height was similar between early/late and average tempo group. The PHV might be greater in the early than in the late maturing group, however the difference was significant only in boys. Further longitudinal studies including pubertal development are needed.

